# Infections in the Developing Brain: The Role of the Neuro-Immune Axis

**DOI:** 10.3389/fneur.2022.805786

**Published:** 2022-02-17

**Authors:** John Kim, Clara Erice, Ursula K. Rohlwink, Elizabeth W. Tucker

**Affiliations:** ^1^Department of Anesthesiology and Critical Care Medicine, Johns Hopkins University School of Medicine, Baltimore, MD, United States; ^2^Center for Infection and Inflammation Imaging Research, Johns Hopkins University School of Medicine, Baltimore, MD, United States; ^3^Center for Tuberculosis Research, Johns Hopkins University School of Medicine, Baltimore, MD, United States; ^4^Faculty of Health Sciences, Neuroscience Institute, University of Cape Town, Cape Town, South Africa

**Keywords:** microglia, astrocyte, development, central nervous system (CNS) infection, neurological sequelae, pediatrics

## Abstract

Central nervous system (CNS) infections occur more commonly in young children than in adults and pose unique challenges in the developing brain. This review builds on the distinct vulnerabilities in children's peripheral immune system (outlined in part 1 of this review series) and focuses on how the developing brain responds once a CNS infection occurs. Although the protective blood-brain barrier (BBB) matures early, pathogens enter the CNS and initiate a localized innate immune response with release of cytokines and chemokines to recruit peripheral immune cells that contribute to the inflammatory cascade. This immune response is initiated by the resident brain cells, microglia and astrocytes, which are not only integral to fighting the infection but also have important roles during normal brain development. Additionally, cytokines and other immune mediators such as matrix metalloproteinases from neurons, glia, and endothelial cells not only play a role in BBB permeability and peripheral cell recruitment, but also in brain maturation. Consequently, these immune modulators and the activation of microglia and astrocytes during infection adversely impact normal neurodevelopment. Perturbations to normal brain development manifest as neurodevelopmental and neurocognitive impairments common among children who survive CNS infections and are often permanent. In part 2 of the review series, we broadly summarize the unique challenges CNS infections create in a developing brain and explore the interaction of regulators of neurodevelopment and CNS immune response as part of the neuro-immune axis.

## Introduction

The development of the central nervous system (CNS) is a tightly regulated complex and dynamic process. The CNS begins to develop in the third week of gestation and continues to mature postnatally into late adolescence and adulthood (1). The critical periods of perinatal and early postnatal neurodevelopment, together with adolescent maturation of the brain, are particularly sensitive to environmental influences. Therefore, it is not surprising that CNS infections represent a significant source of mortality and morbidity in children worldwide (2–5). In children, these infections lead to devastating neurological sequelae, such as motor deficits or cognitive impairment, that can last a lifetime (6–8). Moreover, immunocompromised children are at higher risk of developing more severe CNS infections with poorer outcomes (9). In this review, we explore the idea that key components of the CNS immune response are also regulators of early neurodevelopment, interacting along the neuro-immune axis. Moreover, we provide an overview of current evidence suggesting that this dual role is a key driver of the unique pediatric (0–18 years) susceptibility to CNS infections and the resultant sequelae. Specifically, we highlight how a dysregulated immune response secondary to two CNS infections cause long-term neurological sequelae.

## Blood-Brain Barrier

Innate immunity in the CNS begins at the blood-brain barrier (BBB), a semipermeable barrier consisting of brain microvascular endothelial cells (BMECs), stabilized by astrocytic end-feet and pericytes. BMECs line the cerebral vasculature and form a cellular monolayer via tight junctions (TJs) and adherents junctions. The resulting barrier allows for bidirectional regulation of molecular flow and hinders the hematogenous entry of pathogens and toxins (10). The stage at neurodevelopment where the BBB becomes functional remains unclear. Historically, an immature BBB was considered to contribute to an increased risk of CNS infections in children. This notion can be traced back to early studies of dye-injection experiments and observations of higher cerebrospinal fluid (CSF) protein concentrations in newborn animals (11). Conclusions on BBB maturity drawn from these experiments, however, have since been challenged, and mounting evidence supports the existence of a functional BBB early in embryonic development (11).

Nevertheless, even a “mature” BBB is not impregnable. The predominant cause of meningitis in older infants and children is *Streptococcus pneumoniae*, which interacts with the endothelial receptors polymeric immunoglobulin receptor (pIgR) and platelet endothelial cell adhesion molecule-1 (PECAM-1 or CD31) to invade the CNS (12). The gram-positive bacterium *Listeria monocytogenes*, a leading cause of meningitis in neonates, enters the CNS by triggering endocytosis in host epithelial cells (13, 14) and hijacking infiltrating monocytes (15). The protozoan parasite *Toxoplasma gondii* similarly utilizes this “trojan horse” method of CNS entry via infected dendritic cells and monocytes (16) while also replicating in endothelial cells (17).

Virulence factors which enable host extracellular matrix-pathogen interactions and the resulting neuroinflammatory response can also disrupt the BBB at the molecular level. For example, Group B *Streptococcus* upregulates Snail1 on BMECs which in turn represses the expression of TJ proteins including occludin, zonula occludens, and claudin-5, leading to a leaky BBB (18). Additionally, the parasite *Plasmodium falciparum*, the major cause of pediatric cerebral malaria (CM), does not cross the BBB. Rather, *P. falciparum* binds to endothelial cells, triggering an inflammatory cascade that ultimately leads to BBB damage, vascular leakage, and often lethal cerebral edema (19). Even peripheral infections such as sepsis can lead to neuroinflammation with similar BBB damage, as demonstrated by the absence of occludin on brain autopsies from septic patients (20).

Once pathogens compromise the BBB or/and enter the CNS, the CNS must mount a sufficient immune response to control the infection. Unfortunately, the host immune response may become dysregulated and initiate various injury cascades that compromise normal neurodevelopment.

## At the Junction of CNS Immunity and Neurodevelopment: the Neuro-Immune Axis

**Figure 1:**
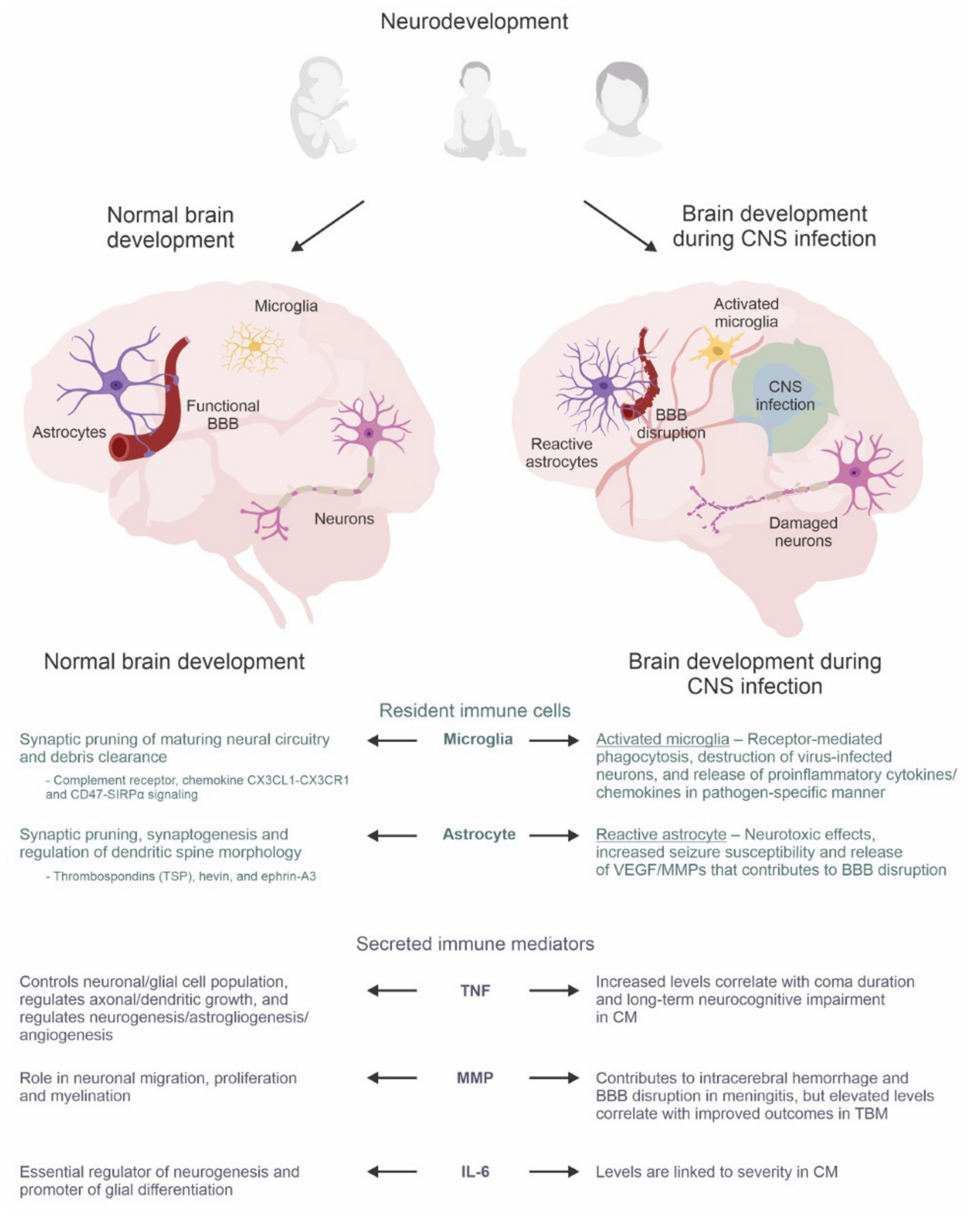
The neuro-immune axis in the developing brain. Astrocytes, microglia, and secreted immune mediators have dual roles during normal brain development and the immune response triggered by CNS infections. During normal brain development, they are integral to synaptic pruning, regulation of dendritic growth/morphology, neurogenesis, astrogliogenesis, angiogenesis, myelination, and glial differentiation. However, amid the immune response during infection, brain development becomes dysregulated which can lead to neurological, neurodevelopmental, and psychiatric sequelae. The lower panel summarizes the roles of resident immune cells (microglia and astrocytes) and secreted immune mediators (TNF super family, MMP, IL-6) during normal brain development compared to dysregulated brain development during CNS infection. Abbreviations: BBB, blood-brain barrier; CM, cerebral malaria; CNS, central nervous system; MMP, matrix metalloproteinases; TBM, tuberculous meningitis; TNF, tumor necrosis factor; VEGF, vascular endothelial growth factors. Created with BioRender.com and CorelDRAW Graphics Suite 2021.

Recent evidence describing dynamic interactions between the CNS and immune system together with the discovery of the CNS lymphatic system (i.e., glymphatic system and meningeal lymphatics) (21, 22) have rendered it necessary to rethink the long-held notion that the CNS is an immune privileged site (23, 24). Instead, it is now suggested that the CNS is a site of active, highly regulated immune surveillance (25). This framework is essential to contextualizing processes whereby microglia, astrocytes, and secreted immune mediators are intimately involved in neurodevelopment (26) (summarized in Figure 1).

Furthermore, the proliferation and maturation of microglia and astrocytes themselves are shaped by complex local and systemic cues which originate from diverse sources, ranging from the developing CNS itself to the maternal microbiome (27, 28). In response to key signals including but not limited to transforming growth factor-β (TGF-β) and colony-stimulating factor-1 (CSF-1), microglia undergo distinct, sequential stages of differentiation with morphological, transcriptomic, and functional transformations (29, 30). Similarly, recent studies have further characterized the temporo-spatial heterogeneity of transcriptomic profiles in astrocytes during perinatal synaptic development (31), circuitry-specific synaptic association (32), and even throughout normal aging (33, 34). Amidst the complex milieu that is the developing CNS, further appreciation of the roles of immunologically active glia and signaling molecules in neurodevelopment is quintessential to understanding the susceptibility of the pediatric CNS to infections and devastating sequelae.

### Microglia

Microglia are resident immune cells of the CNS and an important defense against invading pathogens and tissue injury. Microglial distribution in the brain is heterogenous, ranging from 0.5 to 16.6% of cells in the parenchyma (35). The colonization of the developing brain by embryonic microglia precedes neurogenesis, astrogliogenesis, and vasculogenesis, alluding to the crucial role of microglia in mediating early developmental and homeostatic processes of the CNS (36).

Microglia are dynamic cells that constantly survey their microenvironment and respond to environmental cues. Under pathological conditions they undergo a phenotypic shift from the ramified (or “quiescent”) form to the ameboid (or “activated”) form (37). Their intrinsic role as active surveyors and phagocytes of the brain parenchyma makes microglia key players in synaptic pruning (38–40). Synaptic pruning constitutes the elimination of excess connectivity and its functional structures (i.e., synaptic terminals, axonal and dendritic branches), and is a critical process during the prenatal and postnatal maturation of neural circuitry. Accordingly, depletion of microglia leads to defective pruning in the developing auditory brainstem (41), the visual cortex (42), and the somatosensory cortex (43). To date, numerous studies have identified microglia-specific signaling molecules that underpin the normal development of neural circuitry including complement receptor 3-C3 (44), the chemokine CX3CL1-CX3CR1 (45), and CD47-SIRPα signaling (46).

In order to respond to environmental cues, microglia express extensive molecular tools critical to sampling and interpreting brain milieu. For example, pattern recognition receptors (PRRs) expressed on microglia cell membranes, such as toll-like receptors (TLRs), enable microglia to mount a rapid response to microbial invasion and endogenous cellular damage. These receptors recognize pathogen-associated molecular patterns (PAMPs) and damaged-associated molecular patterns (DAMPs) (47, 48). Moreover, microglia express a cluster of genes, dubbed the microglial “sensome,” that allow them to sense CNS perturbations and include genes encoding purinergic receptors (e.g., *P2RY12, ADORA3, TMEM173*), cytokine and chemokine receptors (e.g., *CSF1R, TGFBR1, IFNGR1, CX3CR1, CMKLR1*), and Fc receptors (e.g., *FCGR3, FCER1G*) (49, 50).

During CNS infections, microglia clear bacteria via receptor-mediated phagocytosis (51, 52), identify and destroy virus-infected neurons (53), and even pave the way for remyelination post-infection by clearing debris and recruiting oligodendrocytes (54). In addition, microglia mediate a broader immune response by secreting key signaling molecules including proinflammatory cytokines and chemokines in a pathogen-specific manner. For instance, microglial activation by lipopolysaccharide (LPS) results in significantly different expression of tumor necrosis factor (TNF) and interleukin (IL)-1α/β compared to stimulation by neurotropic Semliki Forest virus (55). Furthermore, microglia can also cross-present viral antigens from infected neurons to recruit CD8^+^ T cells (56) and phagocytose infiltrating neutrophils to counteract ischemic injury (57).

### Astrocytes

Astrocytes comprise up to 30% of the mammalian CNS. Once thought to be mere scaffolds holding neurons together, astrocytes are now recognized for their dynamic roles in BBB maintenance, neuroinflammation, neurotransmission, and other essential CNS processes (58–60).

Although microglia are important for synaptic pruning, astrocytes are vital to the establishment and fine-tuning of synapses and broader cortical circuitry (61). Therefore, the role of astrocytes during neurodevelopment has primarily been studied in the context of synaptogenesis and synaptic pruning, leading to the identification of various astrocyte-expressed prosynaptogenic molecules (62). For example, astrocytic extracellular glycoproteins called thrombospondins (TSPs) have been characterized to promote excitatory synaptogenesis via interaction with the neuronal α2δ-1 receptor (63, 64). Additionally, the protein SPARCL1 (hevin) is highly expressed by astrocytes during critical periods of early synaptic refinement and is sufficient to selectively induce excitatory synapse formation (65–67). Furthermore, astrocytic ephrin-A3 activates EphA4 receptors on neuronal dendritic spines (DS) and regulates DS morphology and lifetime (68, 69). Notably, many of these molecules are now being examined for their roles during CNS injury and infection. For instance, TSP-1 and TSP-2 are antiangiogenic factors and are upregulated after intracerebral hemorrhage (70). Astrocytic hevin is also pivotal during synaptic remodeling after ischemic injury in an adult stroke model (71). This suggests the sustained importance of astrocytes in synapse integrity beyond perinatal and early postnatal neurodevelopment, though the effect of infection warrants further investigation.

Astrocytes are also indispensable to CNS innate immunity. They constitute an integral component of the BBB, the first line of defense against pathogen invasion (59). Ensheathment of cerebral microvasculature by astrocytic terminal processes (“end-feet”) is critical to BBB integrity (72). While neurotropic pathogens can bypass this barrier, its integrity during infection and inflammation remains a crucial bulwark against pathological leukocyte infiltration and hematogenous entry of additional pathogens. During neuroinflammation and ischemia, astrocytes secrete factors such as vascular endothelial growth factors (VEGFs) and matrix metalloproteinases (MMPs), that contribute to BBB disruption (73–76). Conversely, astrocyte-derived factors including sonic hedgehog can support BBB recovery (77). Therefore, astrocytes play a pivotal role in regulating BBB permeability and can help or harm BBB integrity during brain insults.

Following infection or injury, astrocytes undergo a morphological, transcriptomic, and functional shift through a process referred to as “reactive astrogliosis” (78–80). Reactive astrogliosis is often categorized by the dichotomous A1/A2 system, which many consider too simplistic (80, 81). Typically, A1 astrocytes upregulate proinflammatory genes which mediate neurotoxic outcomes in response to neuroinflammation, and A2 astrocytes are induced upon ischemic injury and favor a neuroprotective transcriptomic profile that promotes tissue repair. The cytokines TNF, IL-1α, and the complement component 1 subcomponent q (C1q), secreted by activated microglia, robustly induce an A1 phenotype (81). Interestingly, the A1 shift is associated with alterations in prosynaptogenic factors (i.e., TSP and hevin) which may prove pathological if it occurs during critical periods of neurodevelopment (81).

Similar to microglia, astrocytes express a variety of PRRs such as TLRs, mannose receptor, and nucleotide-binding and oligomerization domain-like receptors (NLRs) which allow them to respond directly to PAMPs and DAMPs (82). For instance, *Staphylococcus aureus* infection augments TLR2 mRNA expression in astrocytes, and TLR2 knock out mice exhibit attenuated release of TNF and IL-1β (83). Pathogenic activation of astrocytic PRRs and downstream immune signaling pathways may, however, pathologically alter neurodevelopment. Astrocytic TLR4 recognizes LPS, which leads to the expression of TNF, IL-15, and IL-27 through TLR4-MyD88 signaling (84). Early postnatal activation of astrocytic TLR4-MyD88 promotes hippocampal excitatory synaptogenesis and dendritic branching which may underpin increased seizure susceptibility accompanying many CNS infections (85).

### Secreted Immune Mediators

During the CNS immune response to infection, microglia, astrocytes, neurons and BMECs secrete a plethora of cytokines, chemokines and MMPs. The roles of these mediators in innate immunity are well documented, therefore, we will concentrate on their involvement during neurodevelopment. Due to the wide breadth of these secreted factors, we will focus on three classical examples: the TNF superfamily (TNFSF), MMPs, and IL-6.

The TNFSF consists of 19 cytokines which interact with the TNF receptor superfamily (TNFRSF) (86). Today, TNFSF/TNFRSF are known to have variegated roles beyond innate immunity and inflammation. Most members of TNFSF and TNFRSF are constitutively expressed in the mammalian CNS and play non-immunological roles critical to healthy brain development such as neuronal and glial cell population control (87). In addition, they regulate axonal and dendritic growth both in the CNS (88) and peripheral nervous system (89). TNF, the best understood member of this family, regulates neurogenesis, astrogliogenesis, and angiogenesis (90–92). Indeed, TNF^−/−^ mice display reduced numbers of neurons and microglia, and pharmacological TNF inhibition impairs learning and memory in *in vivo* models (93). However, human studies have associated chronic expression of TNF with the development and severity of autism and schizophrenia (94).

MMPs are a family of endopeptidases which mediate broad physiological processes (e.g., bone remodeling, wound healing, embryonic development) via the proteolytic cleavage of extracellular matrix proteins, cytokines, and chemokines. Research into the role of MMPs in the developing brain and spinal cord has revealed their homeostatic and spatiotemporal specific expression, and their roles in neuronal migration, proliferation, and myelination (95–99). Increased levels of MMP-9, one of the most widely studied MMPs, has been linked to various neurodevelopmental disorders; from autism spectrum disorder (100), Fragile X syndrome (101), to schizophrenia (102). In animal models of meningitis, MMP-9 activity has also been shown to contribute to intracerebral hemorrhage and BBB disruption (103, 104). Similarly, elevated MMP-9 and the ratio of MMP-9 to tissue inhibitor of MMP-1 (TIMP-1) have been observed in the CSF of pediatric patients with bacterial meningitis, including tuberculous meningitis (TBM) (105–107). Interestingly, increased MMP-9 during treatment in pediatric TBM patients was associated with improved outcomes, hypothesized to be secondary to MMP's role in neurodevelopment (107).

IL-6 is a cytokine of the interleukin family with both proinflammatory and anti-inflammatory properties that is constitutively expressed at low levels by microglia, neurons, astrocytes, and BMECs (108). In the developing CNS, IL-6 is an essential regulator of neurogenesis (109, 110) and promotor of glial differentiation (111). Notably, neural stem cells are known to self-regulate progenitor pools via autocrine IL-6 signaling, and transient exposure to increased maternal IL-6 is sufficient to dysregulate neural precursor cell pools in the developing forebrain (112). Under pathological conditions, IL-6 levels dramatically increase in the CNS and may be neuroprotective. For instance, microglial IL-6 prevents neuronal loss of neural progenitor cells during herpes simplex virus type 1 infection (113), and endogenous upregulation of IL-6 in response to cerebral ischemia is neuroprotective against excitotoxicity (114). However, elevated IL-6 levels in the fetal brain after maternal immune activation is a key mediator of transcriptional and behavioral alterations in the adult offspring brain (115, 116).

## Neurological Sequelae: A Consequence of the Neuro-Immune Axis?

Both the immune system and neurodevelopment are tightly regulated and versatile processes that must respond to changing microenvironmental cues. Therefore, as outlined above, it is not surprising that they share mechanistic overlap (117) which has left the developing brain vulnerable. It is becoming well accepted that infections during pregnancy and childhood can profoundly affect neurodevelopmental outcome of offspring as well as later in adolescence and adulthood (118). Below we highlight two CNS infections to exemplify how components of these processes are dysregulated and associated with adverse clinical outcomes.

### Cerebral Malaria

CM is one of the deadliest forms of malaria affecting children under 5 years in low- and middle-income countries (LMICs) disproportionately. Despite aggressive antiparasitic therapy and parasite clearance, over 50% of children surviving CM suffer from neurocognitive deficits, seizures, and neurobehavioral disorders (19, 119, 120). Both activated microglia and reactive astrocytes have been linked to the pathobiology of CM in both *in vivo* models and post-mortem human studies (121). The classical proinflammatory cytokines TNF and IL-6 have also been associated with CM outcomes in human studies (122). Specifically, higher CSF levels of TNF correlate with coma duration and long-term neurocognitive impairments (123). Additionally, IL-6 has been linked to severity, where children with CM have increased levels of serum IL-6 compared to those with uncomplicated malaria (124).

### Tuberculous Meningitis

TBM is the most aggressive form of extrapulmonary TB. Like CM, children from LMICs are those most at risk. Pediatric survivors of TBM also suffer from neurodevelopmental deficits in locomotor, personal-social, and language function despite intensive antimicrobial treatment (125, 126). Although numerous factors drive pathogenesis, preclinical and clinical studies suggest the host inflammatory response as a primary contributor to clinical manifestations and sequelae in TBM (127, 128). A study utilizing a murine model showed uncontrolled tissue pathology without the use of treatment targeting neuroinflammation (129), and a rabbit model of pediatric TBM has demonstrated elevated levels of activated microglia in the brains of infected rabbits compared to those of healthy rabbits (130).

Furthermore, a study of South African children with TBM performed transcriptomic analysis and found compartmentalized immune response with increased cytokine signaling in lumbar CSF and increased neuronal excitotoxicity associated with glutamate release in ventricular CSF (131). This glutamate dysregulation within the ventricular milieu may be a manifestation of injury to astrocytes, who are known to be key regulators of glutamate homeostasis (132). In fact, glial fibrillary acidic protein (GFAP), an astrocyte surface marker, along with neuronal injury biomarkers [e.g., S100B and neuron-specific enolase (NSE)] increased in the CSF of pediatric TBM patients with poor outcomes (133). While serum S100B levels fail to reflect the progressive injury in TBM (133), serial measurement of S100B has been proposed to have prognostic value in pediatric patients after traumatic brain injury (134). Hence, sequential evaluation of plasma/serum S100B may prove more informative in TBM.

### Adjunctive Therapies

In both CM and TBM, appropriate antimicrobial therapy does not prevent devastating neurological injury and highlights the need for adjunctive therapy. Currently, there are no approved adjunctive host-directed therapeutics for CM and to date, corticosteroids are the only standard of care host-directed therapy for TBM. Yet, corticosteroids cause side effects and, although they improve TBM mortality, they do not reduce neurological sequelae in survivors and exhibit poor BBB penetration (126, 128, 135, 136). Modulating chemokine and cytokine responses via monoclonal antibodies is an attractive avenue for treating CNS infections. However, these biologics do not cross the BBB and have not shown conclusive results in clinical trials for sepsis and CM (19).

Understanding the dual role that microglia, astrocytes, and secreted immune mediators play during development and the CNS immune response may additionally aid in identifying novel or repurposed drugs to attenuate neurological sequelae. The window of opportunity may also be greatest in children, as they carry the greatest risk of CNS infection and the greatest need to maintain capacity for proper neurodevelopment. Most importantly, any proposed adjunctive therapeutic should aim to have good BBB penetration, and efforts to overcome this bottleneck via novel drug delivery technologies are ongoing (137).

## Conclusion

The developing brain of a child is particularly vulnerable to CNS infections because the cells, molecules and signaling pathways that govern the host response to infection also coordinate key aspects of neurodevelopment. This is evident by the fact that survivors of CNS infections, regardless of etiology, often develop not only neurological disorders but long-term neurological sequelae. Thus, it is imperative to further our understanding of the neuro-immune axis during development to design more effective host-directed adjunctive therapeutics that not only attenuate neuroinflammation but also restore key neurodevelopmental processes.

## Author Contributions

JK, CE, UR, and ET conceptualized the manuscript. JK and CE wrote the initial draft and all coauthors edited the manuscript. ET and UR provided funding. All authors contributed to the article and approved the submitted version.

## Funding

ET is supported by the NIH NIAID K08AI139371 and the Hartwell Foundation−2018 Individual Biomedical Research Award. UR is supported by the Crick African Career Accelerated Award.

## Conflict of Interest

The authors declare that the research was conducted in the absence of any commercial or financial relationships that could be construed as a potential conflict of interest.

## Publisher's Note

All claims expressed in this article are solely those of the authors and do not necessarily represent those of their affiliated organizations, or those of the publisher, the editors and the reviewers. Any product that may be evaluated in this article, or claim that may be made by its manufacturer, is not guaranteed or endorsed by the publisher.
